# A novel immune-related genes prognosis biomarker for hepatocellular carcinoma

**DOI:** 10.18632/aging.202173

**Published:** 2020-11-26

**Authors:** Kunpeng Wang, Xinyi Chen, Chong Jin, Jinggang Mo, Hao Jiang, Bin Yi, Xiang Chen

**Affiliations:** 1 Department of General Surgery, Taizhou Central Hospital (Taizhou University Hospital), Taizhou 318000, China; 2 Department of Anesthesia Surgery, Taizhou Central Hospital (Taizhou University Hospital), Taizhou 318000, China; 3 Department of Cardio-Vascular Surgery, Sun Yat-Sen Memorial Hospital, Sun Yat-Sen University, Guangzhou 510120, China; 4 Department of Anesthesia, The Sixth Affiliated Hospital of Sun Yat-Sen University, Guangzhou 510655, China

**Keywords:** hepatocellular carcinoma, tumor microenvironment, immune-related genes, prognostic, TCGA

## Abstract

Background: Hepatocellular carcinoma (HCC) is closely associated with the immune microenvironment. To identify the effective population before administering treatment, the establishment of prognostic immune biomarkers is crucial for early HCC diagnosis and treatment.

Results: A total of 335 IRGs identified from 788 overlapping IRGs were associated with the survival of HCC. A prognostic immunoscore model was identified. The Kaplan-Meier survival curves and time-dependent ROC analysis revealed a powerful prognostic performance of immunoscore signature via multi validation. Besides, the immunoscore signature exhibited a better predictive power compared to other prognostic signatures. Gene set enrichment analysis showed multiple signaling differences between the high and low immunoscore group. Furthermore, immunoscore was significantly associated with multiple immune cells and immune infiltration in the tumor microenvironment.

Conclusions: We identified the immunoscore as a robust marker for predicting HCC patient survival.

Methods: Three sets of immune-related genes (IRGs) were integrated to identify the overlapping IRGs. Weighted gene co-expression network analysis was performed to obtain the survival-related IRGs. Further, the prognostic immunoscore model was constructed via LASSO-penalized Cox regression analysis. Then the prognostic performance of immunoscore was evaluated. In addition, ESTIMATE and CIBERSORT algorithms were applied to explore the relationship between immunoscore and tumor immune microenvironment.

## INTRODUCTION

Hepatocellular carcinoma (HCC) is one of the most common cancers and the fourth leading cause of cancer deaths in men and women globally, with an estimated 841,000 new cases reported in 2018 [1]. Complete surgical resection remains the standard therapeutic regimen for the early stage of HCC patients, while poor prognosis due to late diagnosis still places a huge burden on individuals and countries. The TNM system and liver functions are commonly used in predicting the survival of HCC patients and determining therapeutic regimens [2]. However, HCC within the same TNM stage might also have a different prognosis because of the inherent clinicopathological and molecular diversities of the disease [3, 4]. Therefore, there is an urgent need to explore a novel approach to guide clinical treatment and improve the prognosis of HCC patients.

Mounting evidence indicates that immunotherapy including the blockade of immune checkpoints has emerged as a potential alternative therapy for HCC [5, 6, 7]. Owing to the overexpression of inhibitory ligands, most tumors evade the immune system by damping the T cell attack [8]. Importantly, the HCC tumor microenvironment is complex and immunogenic, it expresses tumor antigens, and coordinate numerous hepatic antigens presenting cells and thus promotes the evasion of tumor cells from an effective immune response [9]. With the emergence of immune checkpoint therapy including programmed cell death protein (PD-1), and programmed death-ligand 1 (PD-L1), the treatment of advanced HCC patients using a strong response is possible. However, only a minority of HCC patients experience prolonged survival time, with the majority of patients having limited or no response to the therapy particularly during HCC progression. A multi-immune-relevant gene signature that could help clinicians to predict the prognosis of HCC patients and characterize their tumor microenvironment would be highly valuable.

In this study, overlapping immune-related genes (IRGs) from three independent datasets were analyzed and subjected to weighted gene co-expression network analysis (WGCNA) to identify survival-related IRGs. An immunoscore model was constructed using LASSO-penalty regression analysis to predict the survival of patients in three large cohorts. Comprehensive bioinformatics analyses were conducted to explore the underlying mechanisms of the biomarker using ESTIMATE and CIBERSORT algorithms.

## RESULTS

### Identification of survival-related IRGs

**Figure 1:**
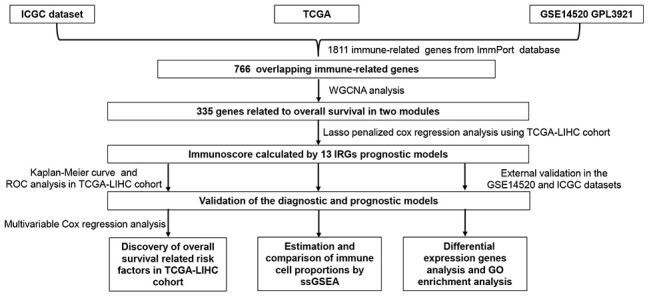
**Flowchart describing the process used to identify and validate the immunoscore of hepatocellular carcinoma.**

**Figure 2:**
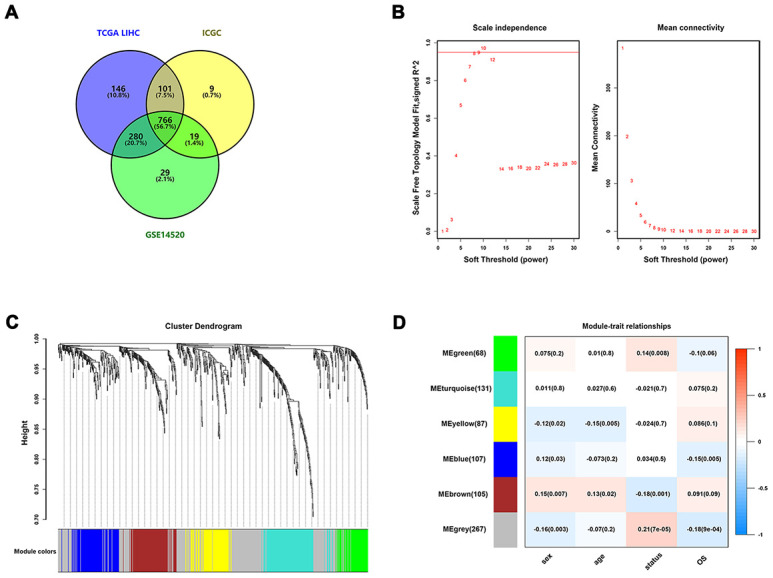
**Identification of survival-related IRGs by using WGCNA.** (**A**) Venn diagram was used to visualize overlapping IRGs among TCGA dataset, GEO dataset, and ICGC dataset; (**B**) The scale-free fit index for soft-thresholding powers. The soft-thresholding power in the WGCNA was determined based on a scale-free R^2^ (R^2^ = 0.95). The left panel presents the relationship between the soft-threshold and scale-free R^2^. The right panel presents the relationship between the soft-threshold and mean connectivity. (**C**) A dendrogram of the differentially expressed genes clustered based on different metrics. Each branch in the figure represents one gene, and every color below represents one co-expression module. (**D**) A heatmap showing the correlation between the eigengene and clinical traits. The correlation coefficient in each cell represented the correlation between gene module and the clinical traits, which decreased in size from red to blue.

The flowchart of the procedures in this study is shown in Figure 1. A total of 788 HCC patients from three independent cohorts were enrolled in our study. After matching the IRGs from the ImmPort database, three sets of data were integrated and 766 overlapping IRGs were identified for further analysis (Figure 2A, Supplementary Table 1).

To construct co-expressed networks and identify co-expression modules, the gene expression profiles of overlapping IRGs were analyzed by WGCNA. Based on a scale-free R^2^ (R^2^ = 0.95) calculated in WGCNA, the soft-thresholding power was determined, and six modules were identified by the soft-thresholding power and the average linkage hierarchical clustering (Figure 2B, 2C). For each module, the gene co-expression networks were summarized in the eigengene. We analyzed the correlations of each eigengene with clinical traits, including sex, age, survival times, and status. As presented in Figure 2D, the green and grey modules were negatively correlated with the survival time and status of HCC patients (*P* < 0.01), whereas the brown module showed the positive correlation with survival status (*P* = 0.001). The green module contained 68 IRGs while the grey module contained 267 IRGs. Data for the two modules were selected for further analysis.

### Construction of prognostic model based on survival-related IRGs

**Figure 3:**
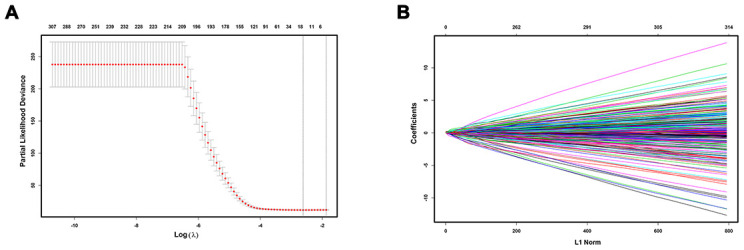
**Construction of immunoscore model.** LASSO deviance profiles (**A**) and LASSO coefficient profiles (**B**).

**Figure 4:**
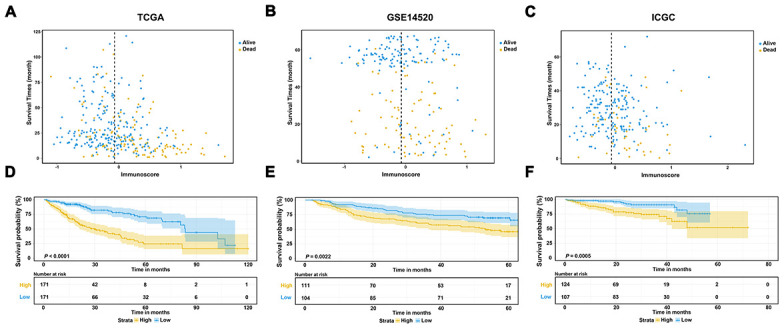
**Evaluation and validation of the prognostic performance of immunoscore in three independent cohorts.** (**A**–**C**) Distribution between the immunoscore and survival data in TCGA dataset (**A**), GSE14520 dataset (**B**), and ICGC dataset (**C**), respectively; (**D**–**F**) Kaplan-Meier survival curves of the immunoscore in TCGA, GSE14520, and ICGC datasets.

**Figure 5:**
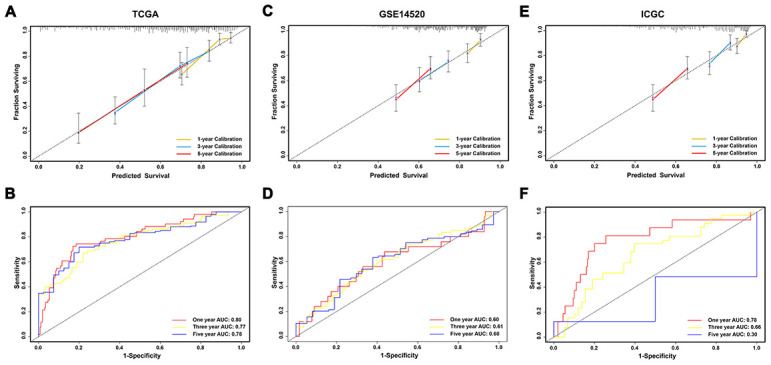
**Evaluation and validation of the prediction value of immunoscore in three independent cohorts.** (**A**, **C**, **E**) Calibration plot of the immunoscore for predicting the probability of survival at 1-, 3-, and 5-years in TCGA, GSE14520, and ICGC datasets, respectively; (**B**, **D**, **F**) Time-dependent ROC curve of immunoscore for 1-, 3-, and 5-year overall survival predictions in TCGA, GSE14520, and ICGC datasets, respectively.

To construct a prognostic model, Lasso penalized cox regression analysis was performed with 335 survival-related IRGs and 13 IRGs were finally identified in the model, consisting of SPP1, STC2, HSPA8, IL15RA, BLNK, TRAF3, NOD2, GRB2, ACTG1, HDAC1, S100A9, PSMD1, and EPO (Figure 3A, 3B). Then the immunoscore was calculated for each patient with the corresponding coefficients (Table 1). The optimal cut-off value was determined by the median value of -0.07 (Figure 4A–4C). Subsequently, the included 342 TCGA-LIHC patients were stratified into two groups based on the optimal cut-off value. The high immunoscore group showed a poor OS compared to the low immunoscore group using the Kaplan-Meier curve analysis (*P* < 0.0001) (Figure 4D). The C-index of the immunoscore was 0.741 (95% CI 0.692 - 0.789) (Figure 5A). Time-dependent ROC curves showed that the area under the curve of ROC (AUC) for the 1-, 3-, and 5- year OS predictions of the immunoscore were 0.80, 0.77, and 0.78, respectively (Figure 5B).

**Table 1:** The IRGs in the immunoscore model associated with OS in the TCGA dataset.

**Symbol**	**Univariate Cox regression analysis**			**LASSO coefficient**
	**HR**	**95%CI**	***P-*value**	
SPP1	1.139	1.080-1.201	1.49e-06	0.0252
STC2	1.382	1.206-1.585	3.29e-06	0.0964
HSPA8	1.569	1.233-1.996	0.000249	0.0763
IL15RA	1.546	1.257-1.903	3.78e-05	0.0673
BLNK	0.711	0.575-0.879	0.001629	-0.0102
TRAF3	1.664	1.222-2.267	0.001237	0.1197
NOD2	1.469	1.141-1.890	0.002833	0.0486
GRB2	2.150	1.437-3.216	0.000195	0.0485
ACTG1	1.525	1.249-1.863	3.54e-05	0.0262
HDAC1	1.933	1.435-2.603	1.43e-05	0.1284
S100A9	1.288	1.172-1.417	1.76e-07	0.0587
PSMD1	2.886	1.815-4.588	7.42e-06	0.2254
EPO	1.285	1.162-1.420	9.35e-07	0.0855

Abbreviations: IRGs, immune-related genes; OS, overall survival; TCGA, The Cancer Genome Atlas; HR, hazard ratio; CI, confidence interval; LASSO, the Least Absolute Shrinkage and Selection Operator.

### External validation of the prognostic performance in the GSE14520 and ICGC datasets.

To validate the classification performance of the immunoscore signature with different data platforms, the GSE14520 dataset and ICGC dataset were used as external datasets. Similarly, each patient obtained exclusive immunoscore and was grouped into two groups based on the optimal cutoff value. The Kaplan-Meier survival curves also showed significant favorable OS in the groups with lower immunoscore score (*P* < 0.01) (Figure 4E, 4F). Moreover, the C-index of the immunoscore in the GSE14520 dataset was 0.596 (95% CI 0.660 - 0.531), while that in the ICGC dataset was 0.697 (95% CI 0.609 - 0.786) (Figure 5C, 5E). The AUCs of the immunoscore model for OS prediction in the GSE14520 cohort was 0.60 at 1 year, 0.61 at 3 years, and 0.60 at 5 years (Figure 5D). In addition, only 2 patients with OS over 5 years in the ICGC dataset were reported, which is not enough for effective ROC analysis. Hence, the AUC for the 5-year OS prediction was unsatisfactory. However, the AUCs for 1- and 3-year OS in the ICGC dataset showed a great diagnostic ability of the immunoscore (Figure 5F). Notably, the immunoscore had better predictive power and accuracy compared to other potential prognostic markers based on time-ROC analysis (Supplementary Figure 1A–1C).

### Correlation with clinicopathological characteristics and prognostic factor

Among the 221 patients enrolled in the TCGA-LIHC cohort with complete clinical information, vascular invasion was found to be significantly correlated with immunoscore (Table 2). Besides, the univariable and multivariable Cox regression analyses indicated that the immunoscore and AJCC stage were both independent prognostic factors for OS (Table 3).

**Table 2:** Correlation of clinicopathologic characteristics and the immunoscore in TCGA LIHC cohorts.

**Characteristics**	**TCGA-LIHC**		
	**Low immunoscore N = 124**	**High immunoscore N = 97**	***P***
Follow-up time (mouths)	35.4 ± 25.5	24.7 ± 20.2	**0.001**
Immunoscore	-0.39 + 0.24	0.31 + 0.34	**0.000**
Age (years)			0.089
< 60	68 (54.8%)	42 (43.3%)	
≥ 60	56 (45.2%)	55 (56.7%)	
Sex			0.488
Female	38 (30.6%)	34 (35.1%)	
Male	86 (69.4%)	63 (64.9%)	
BMI (kg/m^2^)			0.689
< 25	66 (53.2%)	49 (50.5%)	
≥ 25	58 (46.8%)	48 (49.5%)	
G stage			0.123
G1 + G2	73 (58.9%)	47 (48.5%)	
G3 + G4	51 (41.1%)	50 (51.5%)	
Residual tumor			0.873
R0	117 (94.4%)	92 (94.8%)	
Non-R0	7 (5.6%)	5 (5.2%)	
AJCC stage			0.167
I + II	106 (85.5%)	76 (78.4%)	
III + IV	18 (14.5%)	21 (21.6%)	
Vascular invasion			**0.002**
No	92 (74.2%)	53 (54.6%)	
Yes	32 (25.8%)	44 (45.4%)	
AFP (ng/ml)			0.385
< 300	97(78.2%)	71 (73.2%)	
≥ 300	27 (21.8%)	26 (26.8%)	
Total bilirubin (mg/dl)	0.81 ± 0.50	0.94 ± 1.03	0.239

Abbreviations: TCGA LIHC, The Cancer Genome Atlas Liver Hepatocellular Carcinoma; BMI, body mass index; AJCC, The American Joint Committee on Cancer.

**Table 3:** Univariate and multivariate Cox regression analysis of TCGA-LIHC patients.

**Characteristics**	**Univariate Cox**		**Multivariate Cox**	
	**HR (95%CI)**	***P***	**HR (95%CI)**	***P***
Immunoscore	7.372 (3.995 – 13.605)	<0.001	5.917 (3.034 – 11.539)	**< 0.001**
Age (years)				
< 60	1		1	
≥ 60	1.392 (0.819 - 2.367)	0.222	1.057 (0.589 – 1.899)	0.852
Sex				
Female	1		1	
Male	0.648 (0.380 - 1.105)	0.111	0.911 (0.508 - 1.634)	0.755
BMI (kg/m^2^)				
< 25	1		-	-
≥ 25	1.318 (0.779 – 2.229)	0.303	-	-
G stage				
G1 + G2	1		1	
G3 + G4	1.578 (0.931 - 2.676)	0.090	1.204 (0.680 - 2.133)	0.524
Residual tumor				
R0	1		-	-
Non - R0	1.023 (0.247 - 4.237)	0.975	-	-
AJCC stage				
I + II	1		1	
III + IV	2.664 (1.529 – 4.642)	0.001	2.229 (1.242 – 3.999)	**0.007**
Vascular invasion				
No	1		1	
Yes	2.035 (1.182 – 3.505)	0.010	1.433(0.822 - 2.499)	0.205
AFP (ng/ml)				
< 300	1		-	-
≥ 300	1.392 (0.819 - 2.367)	0.222	-	-

Abbreviations: TCGA LIHC, The Cancer Genome Atlas Liver Hepatocellular Carcinoma; HR, hazard ratio; CI, confidence interval; BMI, body mass index; AJCC, The American Joint Committee on Cancer.

### Identification of DEGs between high and low immunoscore group

**Figure 6:**
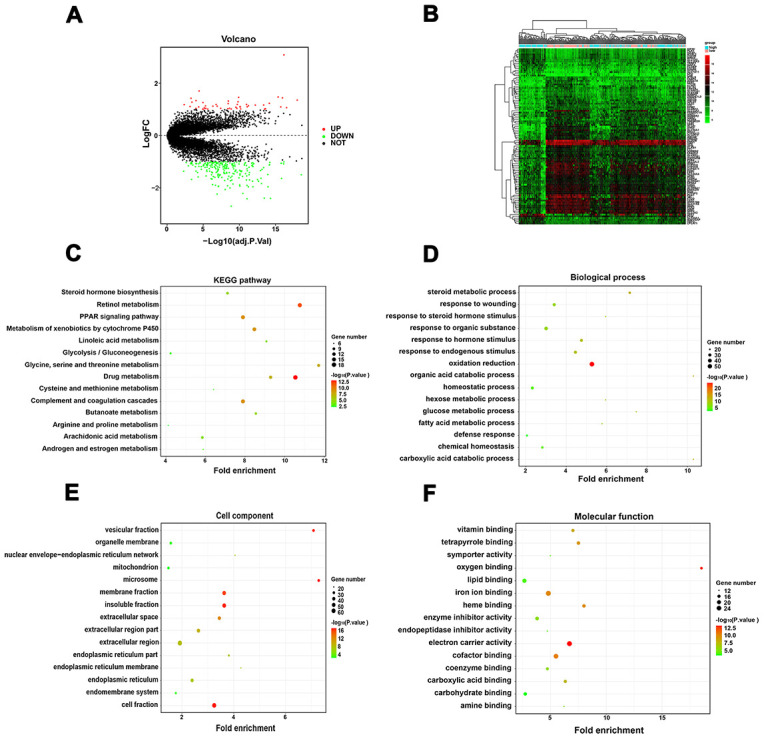
**Identification of DEGs between the low and high immunoscore group and functional analysis.** (**A**) Volcano plot of DEGs in TCGA-LIHC dataset; (**B**) Heatmap of top 50 regulated DEGs in TCGA-LIHC dataset; (**C**) KEGG pathway analysis of the DEGs; (**D**) Biological process of the DEGs; (**E**) Cell component of the DEGs; (**F**) Molecular function of the DEGs.

DEGs analysis between the high and low immunoscore groups in the TCGA-LIHC cohort was performed. A total of 47 upregulated and 225 downregulated genes were identified (Supplementary Table 2). Volcano plot and heatmap were generated to show the distribution of the DEGs (Figure 6A, 6B). Besides, GO enrichment analysis was performed using the online DAVID tool (Supplementary Table 3). KEGG pathway analysis showed that the DEGs were mainly enriched in drug metabolism, retinol metabolism, complement and coagulation cascades and PPAR signaling pathway (Figure 6C). Concerning biological processes, the DEGs were significantly enriched in oxidation reduction, organic acid catabolic process, carboxylic acid catabolic process, and steroid metabolic process (Figure 6D). Enrichment analysis of cellular compartment and molecular functions and the corresponding distributions are shown in Figure 6E, 6F.

### GSEA analysis

**Figure 7:**
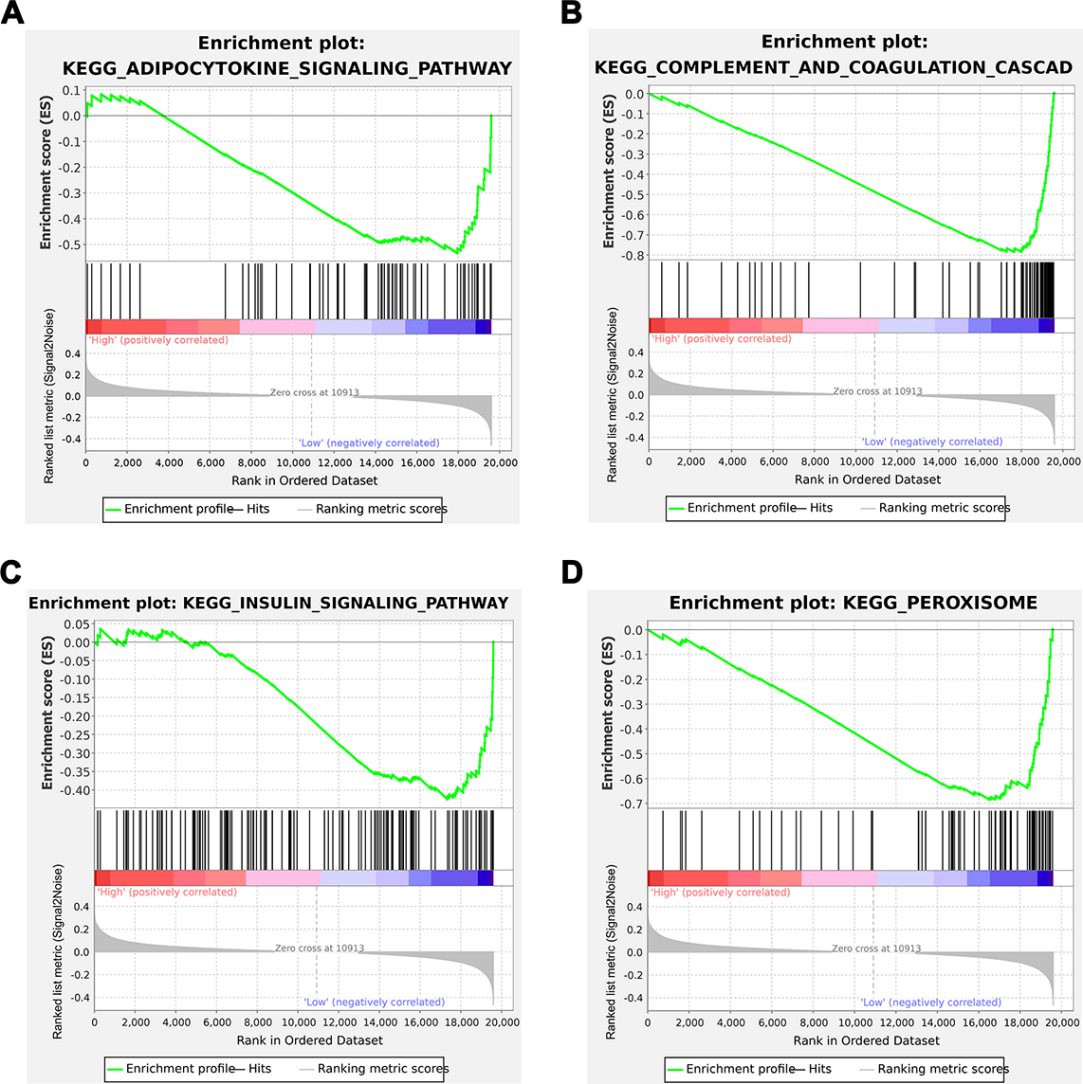
**Gene Set Enrichment Analysis (GSEA) analysis between the low and high immunoscore groups.** (**A**) Adipocytokine signaling pathway; (**B**) Complement and coagulation cascades; (**C**) Insulin signaling pathway; (**D**) Peroxisome.

Gene set enrichment analysis between the high immunoscore and low immunoscore groups was conducted, which showed that 24 significant KEGG pathways were involved, including insulin signaling pathway, peroxisome pathway, adipocytokine signaling pathway, and complement and coagulation cascades pathway, which were identified as immune-related pathways (Figure 7A–7D). GO terms including biological processes, cellular compartment, and molecular functions are listed in Supplementary Table 4.

### Immune infiltration score between the high and low immunoscore group

**Figure 8:**
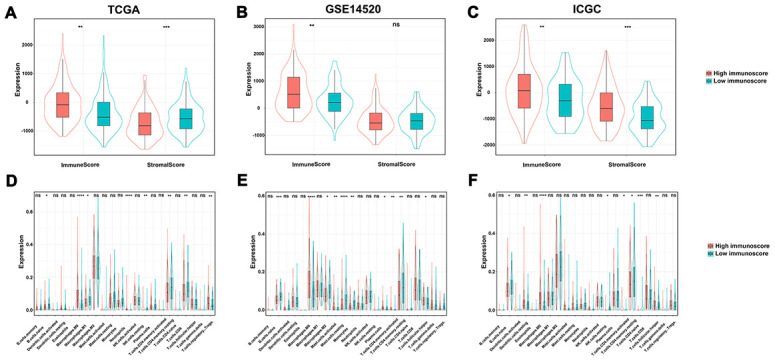
**The relationship among immune score, stromal score and immunoscore.** (**A**) TCGA dataset; (**B**) GSE14520 dataset; (**C**) ICGC dataset. The difference of tumor-infiltrating immune cells among two immunoscore groups. (**D**) TCGA datasets; (**E**) GSE14520 datasets; (**F**) ICGC datasets.

The violin plot shows the relationship between immunoscore with the immune and stromal score. The immune score showed a significant difference between the high and low immunoscore groups despite different results in the different cohorts in terms of stromal score (Figure 8A–8C). Regarding the various immune cells, T cells CD4 memory resting, and B cells naive were significantly aggregated in the low immunoscore group (Figure 8D–8F).

## DISCUSSION

HCC is an aggressive cancer with a high fatality rate and remains a significant global health problem. Although it has long been acknowledged that the immune system regulates tumor initiation and development as well as the targeted therapy strategies, no effective molecular targeted therapy has shown any major impact in routine clinical practice [10–12]. Therefore, due to the limitations associated with immunotherapy, it is necessary to identify effective patients and predict their clinical outcomes. Analysis of immune genes in each patient and prognostic status might solve the current stalemate. In this study, we aimed to establish a valuable prognostic model using immune-related genes, to determine the prognosis of HCC patients.

Only a few prognostic gene signatures have been translated into real clinical practice due to various defects in signature construction. First, the batch effect from a different experimental process rooted in gene expression data always causes nonhomogeneity, which significantly reduces the broad applicability from one specific cohort to another. Second, the robustness and effectiveness of the model are dependent on a large cohort with low variance and independent samples. Besides, the applicability of the calculation method adopted in the model construction and the accurate choice of the statistical method also determine its usability in different populations. Finally, most of the signatures are made up of a few specific genes but ignore the highly heterogeneous genes in HCC, which severely reduces their stability and might cause overfitting.

In this study, we constructed a prognostic model based on IRGs, to reflect the immune status and tumor prognosis in HCC patients. To construct this model, we first screened 765 overlapping IRGs in three independent large cohort. The overlapping IRGs could comprehensively reflect the immune status of HCC. Besides, multiple algorithms were used for model selection, and the prediction value of the model was multi confirmed, which proved the accuracy and dependability of the prognostic model. The expression levels of overlapping IRGs were fitting and grouped into six modules through WCGNA analysis. Ultimately, 13 out of the 335 survival-related IRGs were extracted to construct the prognostic immunoscore model using LASSO regression analysis. Therefore, the HCC patients were grouped into high- or low-risk groups based on the optimal cutoff of the immunoscore. Using the ROC curve and Kaplan-Meier survival curves analyses, the reliability and efficiency of the immunoscore related to survival prediction was evaluated in the TCGA-LIHC cohort and validated using the ICGC and GSE14520 datasets. Univariate and multivariate COX analyses further verified the significant correlation between immunoscore and survival. Furthermore, the CIBERSORT algorithms were used to calculate the immune cell subtype and assess the immune infiltration scores of each sample in the two groups. The results showed that the two immunoscore groups expressed differential immune cell subtypes and survival.

To assess the prediction capability of the model, the immunoscore signature was compared with other prognostic signatures [13, 14]. The immunoscore achieved the highest AUC in 1 year-, 3 year-, and 5 year-ROC analysis, indicating its high prognostic prediction capability. Previous potential gene signatures used differentially expressed genes between HCC and normal tissue to construct a model. A remarkable diagnosis performance was shown, however, these might fail in prognostic performance which ignored that the liver is an organ with predominant innate immunity [15]. It has been recognized that tumor occurrence and progression should be attributed to the internal genetic background of cancer cells and the interaction of the tumor with various systems within the body, specifically the immune system [16, 17]. Immune-related cells and factors regulate the process of hepatocarcinogenesis, proliferation, metastasis, and significantly influence the tumor [18]. Moreover, considering that cancer and immunity are closely associated, the prognostic signature that is expected to aid in clinical treatment should take into account the tumor immune microenvironment. Therefore, the correlation between the IRGs and immune cell infiltration reflected on the status of the tumor microenvironment. The results showed that high immunoscore was significantly correlated with the infiltration levels of B cells, CD4+ T cells, dendritic cells, macrophages, and neutrophils (*P* < 0.05). Previous studies have proven the prognostic function in terms of these immune cells [19–21]. Recently, an extensive immunogenomic study using data compiled by TCGA was performed and included more than 10,000 tumors comprising 33 diverse cancer types [22]. The study identified and characterized six stable and reproducible immune subtypes spanning multiple tumor types by integrating major immunogenomics methods, and also provided a wealth of immune information in different cancer types and the prognostic value. This large-scale study laid a solid foundation for an in-depth understanding of the relationship between tumor and immunity in future studies. Based on the results of this research, we further studied the relationship between liver cancer and immune genes using WGCNA and LASSO analysis and proved that the immunoscore model based on IRGs has a stable and effective prognostic performance. This may provide significant help for future personalized precision treatment for HCC patients.

Additionally, previous studies report that the multi-IRGs in the immunoscore model are linked to tumor prognosis. As a secreted calcium-binding phosphorylation protein, the expression levels of SPP1 have been strongly associated with the expression of the monocyte/macrophage markers CD11b (ITGAM) and CD68, which are significantly associated with lower overall survival. STC2 is a heparin-binding, secreted homodimeric glycoprotein, which is upregulated in T cells developing a Th2 response [23, 24]. It also negatively correlates with immune-related metagenes [25]. HSPA8 is a member of the HSP70 family which also acts as a direct down- regulator of the inflammatory response mediated by DCs and other innate immune system cells [26, 27]. IL15RA is an important component of interleukin-15 (IL15) pro-inflammatory signaling and also interacts with the signals through SYK in neutrophils, and B cell linker protein (BLNK) is also involved with the SYK tyrosine kinase [28–31]. TRAF3 acts as a checkpoint of B cell receptor signaling to control antibody class switch recombination and anergy [32]. Overexpression of Gab2 regulates T-cell receptor (TCR) signaling in Jurkat cells and mediates a feedback inhibitory PI3K signal during TCR activation [33, 34]. High expression of ACTG1 is significantly correlated with advanced tumor stages and poor prognosis in patients with HCC [35]. S100a9 is an intratumoral immunosuppressive cell marker, which maintains tumor progression in the TME including the regulation of antitumor immune cells [36, 37]. The multi-IRGs prognostic signature identified in this study indicated high predictive value and accuracy based on various analyses.

### Limitations

There are inevitably several limitations in this study. First, the gene mapping style released in public available datasets was diverse to avoid the comprehensive inclusion of overlapping IRGs, however this might cause potential error or bias. Secondly, limited clinical information provided by the public database might limit the prognostic capabilities of our model. Preoperative treatment including sorafenib, transarterial chemoembolization, and radiofrequency ablation also contribute to the prognosis of HCC patients, but this data is missing. Thirdly, the three cohorts used to establish immune-based risk models were from countries including Europe, America, and Asia (Japan). Therefore, these findings do not apply to patients from other countries. Finally, more experimental evidence for immunogenomic analysis is essential to verify the roles of immune genes, checkpoint genes, and enriched pathways involved in the immune microenvironment.

## CONCLUSIONS

In conclusion, we identified a multi-IRGs signature with a strong predictive performance. Differences in the OS of high and low immunoscore groups are implicated in immune infiltration, tumor immune microenvironments, and interaction of multiple signaling pathways. The recommended IRGs models provide critical information for advancing the personalized management of HCC patients.

## MATERIALS AND METHODS

### Study population

In this study, three publicly available datasets, including The Cancer Genome Atlas Liver Hepatocellular Carcinoma dataset (TCGA-LIHC) from the Genomic Data Commons (GDC) portal (https://portal.gdc.cancer.gov), GSE14520 dataset (based on the GPL3921 platform) from Gene Expression Omnibus (GEO) website (https://www.ncbi.nlm.nih.gov/geo), and the International Cancer Genome Consortium (ICGC) dataset (https://dcc.icgc.org), were downloaded and analyzed. A total of 371 HCC cases and 50 normal cases were included in TCGA-LIHC dataset, while 342 HCC patients with follow-up times of more than 30 days were included in WGCNA analysis and construction of the gene prognostic models. Besides, 216 and 231 HCC cases from the GSE14520 dataset and ICGC dataset, respectively, were used for external validation of the model. The 221 TCGA-LIHC patients with their complete clinical information and mRNA expression data were included for univariable and multivariable Cox regression analysis.

### RNA-seq and microarray data preprocessing

The gene expression profiling data downloaded from the TCGA database were subjected to normalization with the “Deseq2” package in R [38]. Next, gene annotation was performed using the Ensemble database and log2 transformation was subsequently applied. As for the GSE14520 dataset, multiple probes mapped to a single gene (i.e., unique Entrez gene ID) were determined based on their mean expression values. Probe annotations based on the GPL3921 platform were downloaded from the GEO database. Background correction, quantile normalization of the microarray data followed by log2 transformation for further analysis were conducted. For the ICGC dataset, normalized read count values obtained from the gene expression file were used. Gene expression analysis was performed using Entrez Gene IDs in the three cohorts.

### Immune-related gene extraction

A total of 1,811 IRGs were retrieved from 17 categories after excluding the duplicates from the Immunology Database and Analysis Portal (ImmPort) website (https://www.immport.org) [39]. Regarding the 1,811 IRGs, 765 of them were overlapped in the TCGA-LIHC, ICGC, and GSE14520 datasets. Consequently, the overlapping IRGs were used for further analysis.

### Weighted gene co-expression network analysis

Weighted gene co-expression network analysis for the overlapping IRGs was performed on HCC tissues using the “WGCNA” package in R [40, 41]. From a methodological point of view, WGCNA is divided into two categories, cluster analysis of expression level and phenotypic correlation. WGCNA primarily includes four steps among them: correlation coefficient calculation between genes, determination of gene modules, co-expression network, and a correlation between modules and clinical traits. In brief, the normalized and log2 transformed gene expression data of 342 HCC cases were used to calculate Pearson’s Correlation Matrices. Subsequently, the soft-thresholding power β was set to 8 using the integrated function (pickSoftThreshold) in the “WGCNA” package where the co-expression similarity was raised to achieve scale-free topology. Based on the standard of a mixed dynamic cut tree, the minimum number of genes for each gene module was set to 30, and the IRGs were grouped into 6 modules showing similar expression patterns. A clustering dendrogram was used to display the results of a dynamic tree cut and merging. Eventually, the association between different module genes and the clinical traits was assessed by Pearson’s correlation.

### Immunoscore model construction

The Least Absolute Shrinkage and Selection Operator (LASSO) regression analysis was performed to identify the optimal weighting coefficient of the prognostic IRGs using the “glmnet” package in R [42]. LASSO is considered appropriate for high-dimensional data using penalization and regularization methods for statistical modeling and inhibition of overfitting [43]. Lasso regression performs L1 regularization, which adds a penalty equal to the absolute value of the magnitude of coefficients. This type of regularization can result in sparse models with few coefficients. Some coefficients can be zero and are eliminate from the model. Larger penalties result in coefficient values closer to zero, which are the ideal for producing simpler models. LASSO minimizes the residual sum of squares subject to the sum of the absolute value of the coefficients being less than a constant. The optimal value for penalization coefficient lambda where the partial likelihood deviance is the smallest was determined by running cross-validation likelihood 1,000 times. The λ value was finalized using the lambda.min, which is the value of lambda giving minimum mean cross-validated error. Thus, the immunoscore of each sample was calculated using the following formula: immunoscore = Σexpgenei* βi.

Where expgenei is the expression level of genei, and βi is the coefficient of genei obtained from the LASSO Cox regression analysis in the TCGA dataset. The gene expression value from different datasets should be normalized to fit a relatively uniform scale. Therefore, we transformed the normalized datasets into Z-score to conform to the standard normal distribution using the “scale” package in R. This transformation obtained a uniform underlying distribution (mean = 0, standard deviation = 1) of each gene set across various platforms. The immunoscore was calculated by integrating all Z-score transformed normalized gene expression values with the same formula in the three datasets for further analysis.

### Validation of the immunoscore model

The TCGA-LIHC cohort was used as the training set to evaluate the immunoscore model and contained 342 patients with complete RNA-seq data and survival information. The GSE14520 and ICGC datasets were used to validate the prognostic performance of the immunoscore in HCC patients. The HCC patients were assigned to a high or low immunoscore group based on the cut-off value in the training set. We selected the median value as the cut-off value and also applied the same value in GEO and ICGC datasets to validate the robustness of the model. The Kaplan-Meier survival curve and the C-index were used to compare the predicted and observed overall survival (OS). Univariate and multivariate Cox regression analyses were performed to explore the independent risk factors among immunoscore and other clinical parameters in TCGA-LIHC dataset. Time-dependent receiver operator characteristic (ROC) curve analysis was applied to verify the accuracy and predictive ability of the immunoscore in HCC using the R packages “timeROC” [44]. To evaluate the clinical utility, the immunoscore was also compared with other effective prognostic signatures of HCC [13, 14].

### Comparison of enriched oncogenic pathways

After validating the prognostic value of the immunoscore, analysis of differentially expressed genes (DEGs) was performed between the high and low immunoscore groups using the “Limma” package in R [45]. The thresholds in the absolute value of the log2 fold change (logFC) > 1 and adjusted *P*-value < 0.05 were adopted. A volcano plot was used to visualize the distribution of the DEGs. To detect potential biological functions and involved signaling pathways of immune-related DEGs, functional enrichment analyses including Gene Ontology (GO) and Kyoto Encyclopedia of Genes and Genomes (KEGG) enrichment analyses were performed using DAVID and visualized with the “clusterProfiler” package [46]. GO terms were identified with a strict cutoff of adjusted *P*-value < 0.01 and false discovery rate (FDR) < 0.05. Besides, Gene Set Enrichment Analysis (GSEA) with an adjusted *P*-value < 0.05 was performed.

### Differences in tumor-infiltrating immune cells between groups

To estimate the population-specific immune infiltration, the CIBERSORT program was used to obtain the normalized enrichment scores of each immune category using the R package “cibersort” [47]. The enrichment score represented the degree of absolute enrichment in a gene set in each sample within a given dataset. Normalized enrichment scores were calculated for each immune category with the deconvolution approach application. We included a total of 27 immune cells that are involved in innate immunity among them, CD56dim NK cells, CD56bright NK cells, natural killer (NK) cells, plasmacytoid dendritic cells (DCs), activated DCs, immature DCs, neutrophils, monocytes, eosinophils, mast cells, and macrophages. And in adaptive immunity including, activated B cells, immature B cells, effector memory CD4+ T-, central memory CD4+ T, central memory CD8+ T, activated CD4+ T, effector memory CD8+ T-, activated CD8+ T-, NK T-, T follicular helper, Tγδ, Th1, Th2, Th17, and Treg. A two-sided Wilcoxon test was used to compare the differences in immune cell subtypes in the high and low immunoscore groups.

Infiltrating stromal and immune cells form a major fraction of normal cells in tumor tissue. It perturbs the tumor signal in molecular studies, and promotes cancer biology [48]. The ESTIMATE algorithm, which is described as an estimation of stromal and immune cells in malignant tumors using expression data was used to obtain the immune score and stromal score of each sample using the R package “estimate”.

### Statistical analysis

All statistical analyses were performed using R software (Version 3.6.0; R Foundation for Statistical Computing, Vienna, Austria). Description and comparison of the clinical characteristics of the HCC patients from different immunoscore groups were analyzed using the chi-square test or Fisher's exact test. The two groups of boxplots were analyzed using Wilcoxon-test. Kaplan–Meier survival curves were built using log-rank tests to compare the OS between the two groups. ROC analysis was performed to evaluate the sensitivity and specificity of the survival predicting model based on the immunoscore. All statistical tests were two-sided, *P*-value < 0.05 was considered statistically significant.

### Availability of data and materials

The data included in this study originate from the public free-charged database including The Gene Expression Omnibus (https://www.ncbi.nlm.nih.gov/geo/) and The Cancer Genome Atlas (https://portal.gdc.cancer.gov/).

## Supplementary Material

Supplementary Figure 1

Supplementary Table 1

Supplementary Table 2

Supplementary Table 3

Supplementary Table 4
