# Supplementary material for: A novel immune-related genes prognosis biomarker for hepatocellular carcinoma
Source: Aging (Albany NY). 2020 Nov 26;13(1):675–93. doi: 10.18632/aging.202173 (PMC7834986; doi:10.18632/aging.202173)
Supplement: Supplementary Figure 1 [file aging-13-202173-s001.pdf]

SUPPLEMENTARY FIGURE

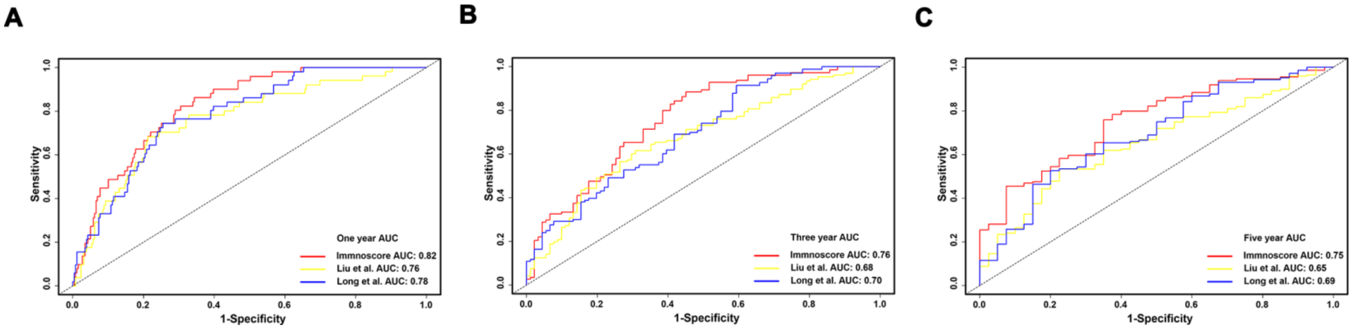

Supplementary Figure 1. The AUC of the immunoscore for 1-, 3-, and 5-year overall survival predictions compared with other studies. (**A**) One year AUC; (**B**) Three year AUC; (**C**) Five year AUC.
